# Experimental Tuberculous Meningitis

**Published:** 1916-09-09

**Authors:** 


### Experimental Tuberculous Meningitis.

Some interesting researches have been conducted at the Johns Hopkins Hospital by Dr. C. R. Austrian dealing with artificially produced tuberculous meningitis in rabbits. From the account given in the *Johns Hopkins Hospital Bulletin* it seems that the introduction of tubercle bacilli into the spinal canal of rabbits leads to the development in those animals of tuberculous meningitis. Various methods were tried to avert the death of a rabbit thus artificially infected with tuberculous meningitis, but without success. Thus albumose-free tuberculin, tuberculinised serum, the serum of tuberculous rabbits, and a suspension of rabbits' leucocytes were all unsuccessfully injected into the spinal canal. So far the results of the investigation were mainly negative; but there is a most valuable suggestion offered at the conclusion of the article which may lead to a new diagnostic criterion of distinct value. Dr. Austrian puts forward the idea that the ready production of meningitis in rabbits by the intraspinous injection of infectious material may be useful in determining the nature of a meningeal infection in man. One or two cubic centimetres of the cerebrospinal fluid of a patient thought to have possibly tuberculous meningitis could be injected into a rabbit's theca, and examination of the rabbit's meninges a few days later might afford valuable evidence as to the nature of the patient's disease.

